# Supplementary material for: Effects of Gestational Age and Early Parenting on Children’s Social Inhibition at 6 Years
Source: Children (Basel). 2019 Jun 28;6(7):81. doi: 10.3390/children6070081 (PMC6678926; doi:10.3390/children6070081)
Supplement: Supplementary file 1 [file children-06-00081-s001.pdf]

Supplementary Material

Table S1

Parent-Infant Relationship Index

Description	Coding
Standardized interview with parents and research nurses' observations of attachment-related parental concerns, feelings, and behavior. All research nurses were trained in advance, but inter-rater agreement was not assessed. The scale comprised 8 items of yes (1) or no (0) ratings on the following items:<br><br>(1) mother does not yet know the infant (mother interview, neonatal),<br>(2) mother visits infant once a week or less (mother interview, neonatal),<br>(3) father visits infant less than once a week (mother or father interview, neonatal),<br>(4) mother is insecure when taking care of child at home (mother interview, neonatal),<br>(5) mother shows little pleasure when interacting with child (nurse observation, neonatal),<br>(6) father shows little pleasure when interacting with child (nurse observation, neonatal),<br>(7) probability that these parents develop problems in taking care of infant is high (nurse observation, neonatal),<br>(8) mother has trouble building a relationship with child (pediatrician rating after mother interview at 5 months of age).<br><br>First, a sum score was calculated by adding one point for each 'yes' answer. Since the resulting sum score did not show a normal distribution as most parents reported and demonstrated a good relationship with their infant, the sum score was recoded into no concerns for the parent–infant relationship (score 0; 52.0%) and some degree of concern for the parent–infant relationship (score 1-8; 48.0%).	PIRI<br><br>(0) no concerns for the parent–infant relationship<br><br>(1) some degree of concern for the parent–infant relationship

Table S2

Social Inhibition Assessment

Description	Coding
The child and mother were placed in soundproof room without toys. The mother sat 1.5 to 2 meters away from the child and was instructed to answer a written questionnaire and not actively engage with the child. When the child started to show signs of being bored (2-5 minutes after entering the room), an adult stranger entered room with a transparent bag filled with toys. The stranger greeted the child and mother, and sat down opposite of the mother about 1 meter from the child. The stranger started unpacking the bag of toys while looking at the child every 10 seconds but not actively approaching the child. If the child had not initiated nonverbal (e.g. pointing to toy and looking at stranger) or verbal (e.g. asking for toy; saying hello) contact after 3 minutes, the stranger asked if s/he wanted to play with the toys. Latencies of child's first (1) nonverbal and (2) verbal social reaction towards the stranger were measured in seconds with a stopwatch and recorded. Latencies were not normally distributed and were thus coded into three categories of social inhibition behavior, based on distribution in healthy (i.e., not neonatally hospitalized) full-term children's (n= 256) responses in the total sample. These cut-offs marked a meaningful difference in social approach behavior (see Table S3).	Nonverbal<br>Disinhibited <180 seconds<br>Normal 180-188 seconds<br>Inhibited >188 seconds<br><br>Verbal<br>Disinhibited <180 seconds<br>Normal 180-227 seconds<br>Inhibited >227 seconds

Table S3

Distribution of Healthy Full Term Control Group[a] (n= 256) Response to Stranger in Social Inhibition Assessment

	n	%	Range s	*M(SD)* s
**Nonverbal**			0-301	158 (67)
Disinhibited <180 s	54	21 %	0-179	41 (53)
Normal 180-188 s	152	59 %	181-188	184 (2)
Inhibited >188 s	50	20 %	189-301	205 (24)
**Verbal**			0-301	178 (82)
Disinhibited <180 s	53	21%	0-179	39 (51)
Normal 180-227 s	152	59 %	181-227	197 (13)
Inhibited >227 s	51	20 %	228-301	268 (29)

Note. s = seconds. [a]The healthy control group included only full term children that were never hospitalized and had no other medical risk.

Table S4

Multinomial Logistic Regression Results for Variables Predicting Non-Verbal Response including Interaction Term (N=1,314)

	Variable	*B*	SE *B*	Wald	df	OR	95% CI
Disinhibited <180 seconds	Intercept	-1.09***	0.29	14.11	1		
	Sex (male)	0.28	0.14	3.76	1	1.32	1.00 – 1.75
	Lower SES	-0.18***	0.05	15.00	1	0.84	0.77 – 0.92
	Lower GA	0.32***	0.07	20.04	1	1.40	1.20 – 1.59
	Good parenting	0.21	0.37	0.32	1	1.23	0.60 – 2.55
	Good parenting * GA	-0.14	0.09	2.06	1	0.87	0.73 – 1.05
Inhibited >188 seconds	Intercept	-1.22***	0.29	17.47	1		
	Sex (male)	-0.35*	0.14	6.50	1	0.70	0.53 – 0.92
	Lower SES	-0.02	0.05	0.25	1	0.98	0.90 – 1.07
	Lower GA	0.26**	0.07	12.11	1	1.29	1.12 – 1.49
	Good parenting	0.39	0.37	1.12	1	1.47	0.72 – 3.01
	Good parenting * GA	-0.11	0.09	1.36	1	0.24	0.75 – 1.08
	LR χ^2	72.68***					

Note: *$p < .05$ **$p < .01$ ***$p < .001$. The reference category is "normal" (180-188 seconds) non-verbal response. GA = gestational age. SES = socioeconomic status.

Table S5

Multinomial Logistic Regression Results for Variables Predicting Verbal Response including Interaction Term (N=1,314)

	Variable	*B*	SE *B*	Wald	df	OR	95% CI
Disinhibited <180 seconds	Intercept	-1.15***	0.29	15.61	1		
	Sex (male)	0.41**	0.14	8.10	1	1.51	1.14 – 2.00
	Lower SES	-0.13**	0.05	7.72	1	0.88	0.81 – 0.96
	Lower GA	0.27***	0.07	13.62	1	1.30	1.13 – 1.50
	Good parenting	-0.01	0.37	0.00	1	0.99	0.47 – 2.06
	Good parenting * GA	-0.10	0.09	1.02	1	0.91	0.76 – 1.09
Inhibited >227 seconds	Intercept	-1.42***	0.29	24.76	1		
	Sex (male)	0.00	0.14	0.00	1	1.00	0.77 – 1.31
	Lower SES	0.16**	0.05	11.65	1	1.17	1.07 – 1.28
	Lower GA	0.09	0.07	1.69	1	1.10	0.95 – 1.26
	Good parenting	-0.25	0.35	0.52	1	0.78	0.40 – 1.54
	Good parenting * GA	0.02	0.09	0.03	1	1.02	0.85 – 1.22
	$LR\ \chi^2$	70.20***					

Note: $^*p < .05$ $^{**}p < .01$ $^{***}p < .001$. The reference category is "normal" (180-227 seconds) verbal response. GA = gestational age. SES = socioeconomic status.
